# A 94-GHz Millimeter-Wave Sensor for Speech Signal Acquisition

**DOI:** 10.3390/s131114248

**Published:** 2013-10-24

**Authors:** Sheng Li, Ying Tian, Guohua Lu, Yang Zhang, Hao Lv, Xiao Yu, Huijun Xue, Hua Zhang, Jianqi Wang, Xijing Jing

**Affiliations:** College of Biomedical Engineering, Fourth Military Medical University, Xi'an 710032, China; E-Mails: shengli@fmmu.edu.cn (S.L.); cyt_bora@sina.com (Y.T.); lugh1976@fmmu.edu.cn (G.L.); yangzhang@fmmu.edu.cn (Y.Z.); fmmulvhao@fmmu.edu.cn (H.L.); yuxiao@fmmu.edu.cn (X.Y.); xinyin.2009@yahoo.com.cn (H.X.); zhanghuajiayuan@163.com (H.Z.); fmmujxj@163.com (X.J.)

**Keywords:** millimeter wave, speech acquisition, radar sensor

## Abstract

High frequency millimeter-wave (MMW) radar-like sensors enable the detection of speech signals. This novel non-acoustic speech detection method has some special advantages not offered by traditional microphones, such as preventing strong-acoustic interference, high directional sensitivity with penetration, and long detection distance. A 94-GHz MMW radar sensor was employed in this study to test its speech acquisition ability. A 34-GHz zero intermediate frequency radar, a 34-GHz superheterodyne radar, and a microphone were also used for comparison purposes. A short-time phase-spectrum-compensation algorithm was used to enhance the detected speech. The results reveal that the 94-GHz radar sensor showed the highest sensitivity and obtained the highest speech quality subjective measurement score. This result suggests that the MMW radar sensor has better performance than a traditional microphone in terms of speech detection for detection distances longer than 1 m. As a substitute for the traditional speech acquisition method, this novel speech acquisition method demonstrates a large potential for many speech related applications.

## 1. Introduction

As commonly used speech acquisition equipment, the microphone suffers from some serious shortcomings such as short detection distance, low directional sensitivity, high susceptibility to acoustic interference noise, and narrow acoustic frequency bandwidth [1]. Therefore, it is necessary to explore new types of speech acquisition methods.

Contact-type speech acquisition sensors, a type of non-acoustic microphones such as a bone-conduction microphone [ 2] and a throat microphone [ 3], can be used to detect strong voices. However, these contact-type sensors inevitably make users uncomfortable and tense and even cause allergies, as well as restrict their activity. For the non-contact type speech acquisition sensors, infrared rays, light waves, and lasers are also used to acquire speech signals by detecting the vibration of a human body or the resonance of objects. Optical sensors allow acquisition of speech/acoustic signals with a high degree of precision, however, they also suffer from unfavorable atmospheric conditions, incompatible composition and properties of objects, optically reflecting surfaces, and surface scattering [ 4]. Therefore, to overcome these limitations associated with optical sensors, millimeter-wave (MMW) frequencies, commonly designated as the frequency range between 30-GHz and 300-GHz (from the 10 mm to 1 mm wavelengths), have been employed for speech detection at longer electromagnetic wavelengths.

The theory and mathematical model of a Doppler radar speech detection method can be found in our previous paper [ 1]. If an unmodulated continuous-wave (CW) radar signal is transmitted toward a human throat where it is phase-modulated by physiological movement, the phase-shifted reflected signal can be captured by a radar receiver and can be down-converted to a baseband signal. Because this baseband signal has been demonstrated to be linearly related to the phase shift of the radar-received signal [ 1], it can be converted into an audible speech signal.

Ka-band MMW CW radars were developed in previous research for speech detection (the Ka-band spectrum spans from 26-GHz to 40-GHz, which corresponds to the 11.5 mm to 7.5 mm wavelength). Li demonstrated that a 40-GHz MMW CW radar can detect and identify exactly the existential speech signals in free space from a speaking person. He also explored the principle of detection of acoustic wave signals based on the wave-propagation theory [ 5]. Li *et al.* [ 6] reported that a typical Ka-band (34-GHz, 8 mm) MMW radar can also be employed to detect speech signals in free space, however, the speech was corrupted by harmonic noise, electrical-circuit noise, and channel noise. Therefore, they also proposed some speech enhancement algorithms to increase the intelligibility/audibility of the radar speech, such as the adaptive wavelet packet entropy algorithm [ 1], perceptual weighting algorithm [ 6], nonlinear spectral subtraction method [ 7], and auditory masking method [ 8]. To extend the speech detection distance, and to increase the speech quality, a superheterodyne receiver was also designed to mitigate the severe DC offset problem and the associated 1/f noise. Other related research activities on radar and speech concentrated on the sensors [ 9– 11] and speech articulator motions [ 12].

Compared with the Ka-band range, the frequency in the W-band range (75–110 GHz) is higher. As a frequency band that exists between the microwave and far-infrared frequencies, the W-band range provides a good tradeoff between range and sensitivity. A representative frequency in this band is 94-GHz, which has been developed for remote monitoring of the human heart and respiration rate [ 13– 15]. A high carrier frequency can be used to overcome certain limitations associated with low frequencies: (1) short wavelengths provide greater sensitivity to small displacements. According to [ 4], the 94-GHz frequency can resolve displacements at a micrometer-level resolution. It is demonstrated that the 94-GHz carrier frequency can detect displacements of as small as 5 μm at a distance of 15 m (>40 ft) and can clearly resolve a 20 μm displacement at a distance of 50 m (≈150 ft) under S/N > 2 conditions. Further, according to Equation (3) below, for a 1 mm vital displacement, the phase variation of the 34-GHz frequency is 81.6° whereas that of the 94-GHz frequency is 225.6° or a 2.76 times more sensitivity than the 34-GHz system. This difference indicates the clear benefit of using the W-band wave; (2) the human skin penetration depth has been estimated to be 0.37 μm at 94-GHz [ 13], and the human dermal thickness is between 2 mm and 3 mm, meaning that almost no signal can penetrate the human skin, and almost all incident waves will be reflected, indicating that the reflected-wave intensity is greater in high frequency; (3) the 94-GHz carrier frequency, as well as the selected waveguide band (WR-10), is within the MMW atmospheric transmission window [ 16], which allows remote detection of subtle physiological movements with small propagation losses over long distances; (4) for an equivalent antenna size, the higher frequency of operation creates a smaller beam angle [ 13]; the smaller the beam width is, the higher is the directivity of the radar antenna. This condition indicates that a high frequency can maintain a collimated beam over a large distance, and prevent multipath propagation, thus allowing accurate measurements to be taken from larger distances; (5) although a higher carrier frequency (such as the 228-GHz system [ 17]) can offer a higher displacement resolution, 94-GHz is still currently considered as the upper frequency limit because MMW mixers and low-noise amplifiers can be manufactured without facing major engineering challenges [ 13], which means that the 94-GHz radar system is economical and practical; (6) the antenna size decreases with the increase in the carrier frequency. At the 94-GHz frequency, we can integrate the antenna into a monolithic MMW integrated circuit, which allows for systems with very small size, that would be very convenient for a wide range of application; (7) compared with optical sensors, the 94-GHz MMW system has a wider detection range of viewing angles. In addition, it can be easily aligned. Therefore, 94-GHz is an attractive frequency for detecting speech signals.

The null-point problem commonly exists in Doppler radar because a null point can be encountered in every quarter wavelength from the radar to the subject, which significantly degrades the detection accuracy [ 18]. Further, the shorter the wavelength is, the closer the distance between adjacent null points is, which means that the null-point problem is more severe for higher carrier frequencies. Two methods are available to resolve the null-point issue. One is the In-phase and Quadrature (I/Q) quadrature receiver method, and the other is the double sideband transmission method. The I/Q demodulation method is more commonly used as it can not only be used to avoid blind phases in sampling, but also improve the signal-to-noise ratio (SNR) by 3 dB compared with the one-signal channel [ 19].

The primary goal of the present study was to demonstrate the feasibility of remote detection of speech signals at relatively large distances using the MMW system. Based on our two previous Doppler radar speech acquisition systems (8 mm MMW and 8 mm superheterodyne systems), a 94-GHz MMW I/Q radar system (3 mm) is employed for speech acquisition. This system has the following features: (1) It uses a superheterodyne receiver. The transmission frequency is 94-GHz, and the frequency of the first balanced mixer is 86.7-GHz. This two-step indirect-conversion transceiver can be used to avoid the severe DC offset problem and the associated 1/f noise at the baseband, which occur normally in direct-conversion receivers; (2) the transmitting and receiving circuits are separate and employ two antennas, which can reduce interference from other directions and increase the detection range. Furthermore, the angle and the distance between the two antennas can be easily adjusted because the transmitting and receiving circuits are separate. The purpose of this study is to evaluate the ability of the 94-GHz MMW radar system to detect speech signals as well as to compare its ability with those of our two previous radar systems (34-GHz). The acquired speech is evaluated by both objective and subjective speech quality evaluation methods. The other performance characteristics such as penetration ability and detection distance are also compared and discussed. On the basis of the objective and subjective evaluations of the speech quality and the frequency-difference comparison, the ability of these three systems in detecting speech signals is compared and discussed.

## 2. Method

### 2.1. Quadrature Doppler Radar Theory

For the 94-GHz CW I/Q Doppler radar system, it transmits a single-tone signal:

(1) $$T(t)=A_{T}(t)\cos(2\pi \;ft+\varphi (t))$$

where *A_T_*(*t*) is the oscillation amplitude, *f* is the oscillation frequency, *t* is the elapsed time, and *φ*(*t*) is the phase noise of the oscillator. If this signal is reflected by the human throat and chest with a phase shift *φ*(*t*), the output of the radar quadrature mixer can be expressed as follows [ 20]:

(2) $$\begin{array}{l} R_{I}(t)=A_{I}(t)\cos(\frac{4\pi x(t)}{\lambda_{0}}+\Delta \varphi )\\ R_{Q}(t)=A_{Q}(t)\sin(\frac{4\pi x(t)}{\lambda_{0}}+\Delta \varphi ) \end{array}$$

where *A_I_*(*t*) and *A_Q_*(*t*) are the amplitudes in Channel I and Channel Q. Δ*φ* represents the phase shift of radar wave propagation and system phase noise. 4*πx*(*t*)/*λ_0_* is the phase where the time-varying displacement information is modulated, in which *x*(*t*) is the target time-varying displacement information. Here for 94-GHz radar wave in free-space, wavelength *λ_0_* is 3.19 mm.

The displacement information *x*(*t*) is clearly modulated on the phase of the signal, therefore, it can be extracted by an arctangent function:

(3) $$\theta (t)=\arctan\frac{R_{I}(t)}{R_{Q}(t)}=\frac{4\pi x(t)}{\lambda_{0}}+\Delta \varphi$$

Because Δ*φ* is constant, the movement *x*(*t*) is linearly proportional to the demodulated result *θ*(*t*). Therefore, the throat displacement *x*(*t*) and amplitude could be calculated as:

(4) $$\begin{array}{l} x(t)=\frac{\lambda_{0}}{4\pi }(\theta (t)-\Delta \varphi )\\ A_{R}(t)=\sqrt{A_{I}^{2}(t)+A_{Q}^{2}(t)} \end{array}$$

### 2.2. The 94-GHz MMW Radar System

**Figure 1.**
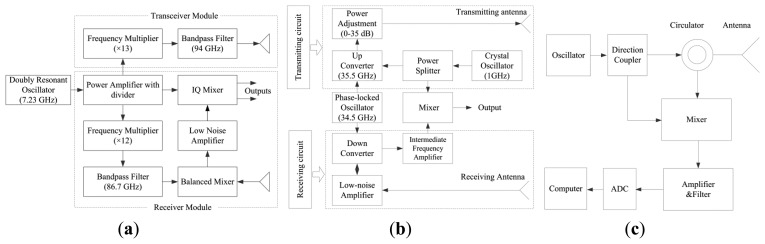
Schematic diagram of the three MMW radar speech acquisition systems. (**a**) The 94-GHz MMW radar system; (**b**) The 34-GHz superheterodyne radar system [ 1]; (**c**) The 34-GHz simple radar system [ 6].

A 94-GHz MMW radar system (ELVA-1 Company, St. Petersburg, Russia) was employed in this study. The schematic of this system is shown in Figure 1a. A W-band double resonant oscillator generated a stable local frequency at 7.23-GHz. The power of the reference frequency was 20 mW, and the frequency stability was 1 × 10^−6^ 1/°C. This frequency was amplified and fed into both the transmitting and the receiving circuits. In the transceiver module (dashed box shown in Figure 1a), the local frequency was multiplied 13 times, followed by a 94-GHz bandpass filtering, and was then fed to the transmitting antenna. In the receiver module (dashed box shown in Figure 1a), the local frequency was multiplied 12 times, followed by an 86.7-GHz bandpass filtering, and was then balance-mixed with the signal from the receiving antenna. After the low-noise amplification, the reflected MMW signal was then homodyne-down-converted by mixing with the local frequency in the IQ mixer. The transmitting and receiving antennas are both Cassegrain antennas with a diameter of 200 mm (ECA-W-10-200, ELVA-1 Company, St. Petersburg, Russia), a gain of 41.7 dB, and a beam angle ∼1°.

As shown by the dashed box in Figure 1a, the transmitter and receiver modules, which are also individual building blocks in the hardware, are relatively independent. This radar-component layout offers the following advantages: (1) it employs a two-step indirect-conversion, thus mitigating the severe DC offset problem that occurs normally in direct-conversion receivers; (2) the two step indirect-conversion can also reduce the associated 1/f noise at the baseband [ 18]; (3) the distance and the angle between the two antennas can be easily changed as the transmitter and receiver modules are two separate blocks. Two other kinds of radar systems, which were used in our previous studies for speech acquisition (8 mm simple radar system and 8 mm superheterodyne radar system) [ 1,8], are also shown in Figures 1b,c for comparison (the readers are referred to the Discussion section for details).

### 2.3. Experiment

#### 2.3.1. Speech Acquisition Experimental System

**Figure 2.**
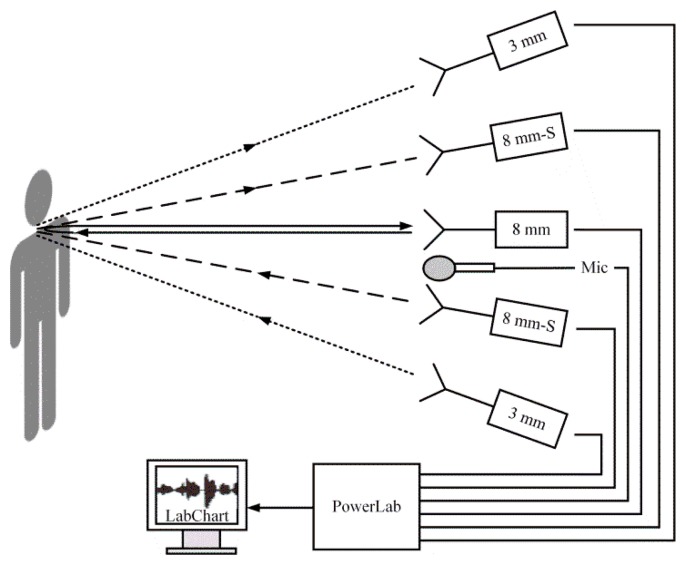
Diagram of experimental system for speech signal detection. The experimental system includes a 3 mm radar system, a 8 mm simple radar system, a 8 mm superheterodyne radar system, and a microphone.

Three measurement radar systems and a microphone were used in this study to *synchronously* detect speech signals, as shown in Figure 2. The distance between the subject and the measurement system was 2 m. A PowerLab 16/35 data-acquisition system (ADInstruments Pty Ltd., sydney, Australia) was used to collect and amplify the signals for the subsequent 16-bit analog-to-digital (A/D) converter. The acquired signals were then processed and analyzed using LabChart (AD Instruments) and Matlab.

#### 2.3.2. Subjects and the Experiment

Ten healthy male volunteer speakers were selected for the speech detection experiments. Their age varied from 24 to 40 years, with a mean age of 31.3 years (SD = 5.15). A volunteer stood in front of the measurement system with his throat at the same height as the radar antenna and 2 m away from the measurement system. Ten Mandarin (Chinese) sentences were selected as speech material for the volunteers. The lengths of the sentences varied from 6 to 13 words (2–11 s). Each participant spoke the sentences in a quiet experimental environment. All experiments were conducted in accordance with the terms of the Declaration of Helsinki (BMJ 1991; 302: 1194), and appropriate consent forms were signed by the volunteers.

#### 2.3.3. Speech Enhancement Algorithm

The quality of speech detected by both the radar systems and the microphone in this study was degraded because of the effects of electrical-circuit noise, harmonic noise, channel noise, and ambient noise. Therefore, a noise-driven short-time phase-spectrum-compensation enhancement algorithm was employed in this study because it can be used under various noise conditions and has been demonstrated to work favorably [ 21].

If *y*(*n*), the noisy speech, consists of the clean speech signal *s*(*n*) and the uncorrelated additive noise signal *d*(*n*), then:

(5) y(n)=s(n)+d(n)

The discrete short-time Fourier transform (DSTFT) of Equation (5) can be represented as:

(6) Y(n,k)=S(n,k)+D(n,k)

where *k* denotes the *k*th discrete-frequency of *N* uniformly spaced frequencies, and the *Y* (*n*,*k*), *S* (*n*,*k*) and *D* (*n*,*k*) are the DSTFTs of noisy speech, clean speech and noise, respectively. Further, the DSTFT of the noisy speech can be written as their magnitude spectrum and the phase spectrum in polar form:

(7) $$Y(n,k)=|Y(n,k)|e^{j∠Y(n,k)}$$

In the proposed speech enhancement algorithm, the noisy magnitude spectrum is recombined with a changed phase spectrum to produce a modified complex spectrum. The noisy complex spectrum is offset by an additive real-valued frequency-dependent ∧(*k*) function:

(8) Y(n,k)=Y(n,k)+Λ(k)

In this study, we employ a simple anti-symmetric function as:

(9) $$\Lambda (k)=\{\begin{array}{ll} \lambda , & if\;0<k/N<0.5\\ -\lambda , & if\;0.5<k/N<1 \end{array}$$

where *λ* is a real-valued constant, and *N* is the length of frequency analysis.

For synthesis, low energy components of the modified complex spectrum cancel out more than the high energy component, thus reducing background noise. For more information about the algorithm, the readers are referred to [ 21].

#### 2.3.4. Speech Perceptual Evaluation

As a subjective measurement method, the mean opinion score (MOS) was used in this study for comparison purposes among the two 34-GHz radar speech, 94-GHz radar speech, and microphone speech. MOS has a five-point scale (1: bad (very annoying); 2: poor (annoying); 3: common (slightly annoying); 4: good (perceptible but not annoying); and 5: excellent (imperceptible)). Ten listeners were selected for the perceptual evaluation. All listeners were native speakers of Mandarin Chinese, had no reported history of hearing problems, and were unfamiliar with the MMW radar speech. The listening tasks were conducted in a soundproof room, and the speech samples were presented to the listeners at a comfortable loudness level (60-dB sound pressure level) via a high-quality headphone. A 4-s pause was inserted before each recited sentence, and the order in which the speech samples were presented was randomized to allow the listeners to respond and to avoid rehearsal effects.

## 3. Experimental Results

**Figure 3.**
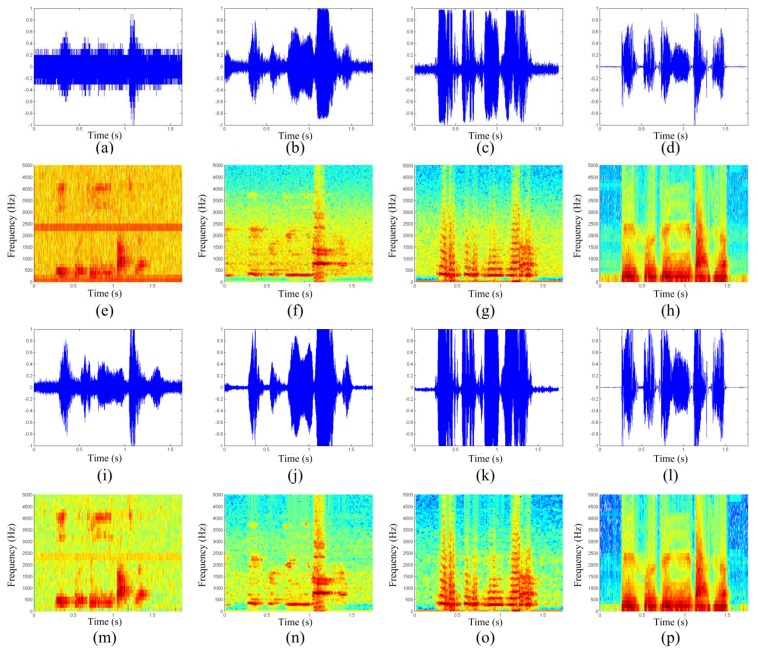
The time-domain waveform and the spectrogram of a typical speech material. The four figures of each column are corresponding to microphone speeches (**first column**), 34-GHz simple radar speeches (**second column**), 34-GHz superheterodyne radar speeches (**third column**), and the 94-GHz quadrature superheterodyne radar speeches (**fourth column**). The first two rows are corresponding to the original speeches, the other rows are corresponding to the enhanced speeches.

Figure 3 shows the time-domain waveform and the spectrogram of the microphone speech (Figure 3a,e,i,m), the 34-GHz simple radar speech (Figure 3b,f,j,n), the 34-GHz superheterodyneradar speech (Figure 3c,g,k,o), and the 94-GHz quadrature superheterodyne radar speech (Figure 3d,h,i,p). Figure 3a–h show the speeches before enhancement, and Figure 3i–p show the corresponding enhanced speeches. Most of the noise signals exist in the time and frequency domains of the microphone signals, even in the enhanced microphone signals, suggesting that the 2 m testing distance is obviously too far for the microphone. In contrast, the noise in the radar speech is less than that in the microphone speech, suggesting that the ability of the radar to detect speeches is stronger than that of the microphone, especially for long distances.

Figure 3 also shows less noise in the 94-GHz radar speech, both in the time-waveform and the spectrogram, suggesting that the 94-GHz radar is immune to acoustic noise because the 94-GHz radar beam is more concentrated than that of the 34-GHz radars. The 94-GHz radar has a beam angle of ∼1°, whereas the 34-GHz radar has a beam angle of 9°. The concentrated beam has higher directivity, which is the reason why the high frequency radar speech has lesser noise. In addition, as the detection frequency becomes higher, the phase variation of the reflected signal increases, which increases the sensitivity to small displacements. Thus, the 94-GHz radar has high sensitivity; hence, the high frequency MMW has a high ability against acoustic interference.

Figure 3 shows that the two 34-GHz radar systems both have higher ability to detect speeches than the microphone. The superheterodyne receiver gives a better performance because it can mitigate the DC offset problem and the associated 1/f noise.

The speech subjective perceptual evaluation (MOS) was used to evaluate the quality of the microphone and the radar speeches. The 400 original sentences (100 microphone speeches, one hundred 34-GHz radar speeches, one hundred 34-GHz superheterodyne radar speeches, and one hundred 94-GHz radar speeches), as well as the corresponding enhanced speeches were rated by 10 listeners. The averaged results are shown in Table 1. The 94-GHz original radar speeches obtains the highest score, followed by the 34-GHz superheterodyne radar speeches and the 34-GHz radar speeches.The microphone obtained the lowest score. This result was also true for the enhanced speeches.

Table 1 shows that the short-time phase-spectrum compensation enhancement algorithm affects the improvement in the speech quality. The average improved score is 0.29. This effect, however, is different for different kinds of speech. The microphone speech obtains a large enhancement effect (0.55 score), but the effect is limited for the 94-GHz radar speech (0.16 score), suggesting that more noise exists in the microphone speech, but less in the 94-GHz radar. These results show that the 94-GHz radar presents the highest speech intelligibility and obtains the best audio quality.

## 4. Discussion

### 4.1. Comparison of the Three Radar Systems

The advantages of the zero-IF radar system are its low power consumption and simple radio architecture. In addition, because it has only one antenna, it can operate down to the zero range. The disadvantage of this kind of radar is the lack of isolation between the transmitting and receiving antennas, which introduces a flicker noise (1/f noise) as well as increase clutter due to the homodyne. Therefore, the desired signal may be masked by noise because the Doppler frequency usually falls in the audio frequency range, which is more susceptible to the flicker noise.

The superheterodyne receiver with non-zero IF can overcome the disadvantage of the zero-IF radar because it uses a two-step indirect conversion, thus increasing the sensitivity and efficiency. The separate antennas can also increase the isolation (reduce the leakage) between the transmitting and receiving antennas.

The 94-GHz radar has a higher detection sensitivity than the 34-GHz radar because flicker noise is inversely proportional to the frequency. It has a non-zero IF, thus possessing all the advantages of the 34-GHz radar system. It has also an I/Q quadrature demodulator receiver, which can be used to avoid the null point. These advantages enable the 94-GHz radar to obtain satisfactory speech detection ability.

### 4.2. Detection Range and Penetrability

Radar can be used to detect speech from a longer distance as compared with a microphone. For example, using a microphone to detect speeches 2 m away is difficult; however, the task is easy for radar from 2 m away. Our experiment shows that the longest distance for acceptable speech quality for the 34-GHz radar is 10 m whereas that for the 94-GHz radar is almost 15 m.

Radar sensors have some penetration ability in that they can penetrate any non-metal media such as clothes and wood. Further, the higher the detection frequency is, the lower is the penetrability, which is in contrast to speech detection: the higher the desired speech quality is, the lower is the penetrability and the shorter is the allowed detection distance. In this study of radar sensors, to maintain speech quality and detection distance, low penetrability, such as in clothes and in thin woods, is allowed for the 34-GHz radar but not for the 94-GHz radar.

### 4.3. High Directivity

Compared with the microphone, the radar system has high directivity, which means that it can be used to directly detect speeches. In addition, for a given antenna size (antenna aperture), the higher the frequency is, the higher is the directivity. An interesting experiment was carried out using a 94-GHz radar. Two persons stood in front of a radar 2 m away with their shoulders very close. The radar was used to detect one of their speeches when they simultaneously spoke different sentences. The results show that the desired sentence was detected and was not disturbed by the other speaker, which is impossible for the microphone. This result suggests that radars can highly detect speeches with high directivity.

### 4.4. Safety

The transmission power of all radars used in this study is only 100 mW. This power is almost 1/10 of the power of a normal cellphone. Further, high frequency emissions can be absorbed by the moisture of the human body. For the 94-GHz radar frequency, the skin depth of a human skin is only 0.37 μm [ 13], which prevents penetration beyond the outer layer of the skin. The 60-GHz frequency has been said to be comparable with exposure to sunlight but at 1/10,000 only of the energy [ 22]. The effect of the 94-GHz may even be lower than this energy.

### 4.5. Applications

The proposed radar sensor may provide a new way or a new sensor/equipment for speech signal detection, which can not only be used as a substitute for existing speech acquisition methods but also compensate for the shortcomings of the traditional microphone speech. These shortcomings include short acquisition distance, low directional sensitivity, and high susceptibility to acoustic noise disturbance. Furthermore, the high frequency MMW radar equipment can be combined with the traditional speech acquisition equipment and can alternatively or synchronously detect speech signals. The radar can be used to detect speeches where the microphone cannot detect. The radar speech can also be combined with the microphone speech to improve the latter's quality. In conclusion, high frequency radar is a novel speech acquisition sensor that can extend the speech and acoustic signal acquisition method to a large extent.

## 5. Conclusions

In this study a 94-GHz radar sensor with superheterodyne and quadrature receivers has been used for speech acquisition. Two 34-GHz radar sensors and one microphone are also used to synchronously detect speeches for comparison purposes. A short-time phase-spectrum-compensation enhancement algorithm is also employed to enhance the detected speech. The speech quality is subjectively evaluated using MOS. The results suggest that the 94-GHz radar sensor obtains the highest speech quality with the highest sensitivity, and it can prevent acoustic disturbance. The superheterodyne quadrature receiver can also mitigate the DC offset problem and the associated 1/f noise. The speech acquisition sensor also possesses some special advantages such as long detection range, less penetrability, high directivity, and safety. Therefore, this novel radar sensor is expected to have a high potential application.

## Figures and Tables

**Table 1.** Speech perceptual evaluation (MOS score) for speeches before and after enhancement.

**Radar System**	**Averaged MOS Scores of Original Speeches**	**Averaged MOS Scores of Enhanced Speeches**
94-GHz	2.56	2.72
34-GHz-S	2.32	2.53
34-GHz	2.11	2.36
Microphone	1.52	2.07
